# Global Salivary Proteomic Profiling of Individuals with the Co-Occurrence of Type 2 Diabetes Mellitus, Dyslipidemia, and Periodontitis

**DOI:** 10.3390/ijms262411778

**Published:** 2025-12-05

**Authors:** Raquel Mantuaneli Scarel-Caminaga, François Isnaldo Dias Caldeira, Sâmia Cruz Tfaile Corbi, Diego Girotto Bussaneli, Alliny de Souza Bastos, Silvana Regina Perez Orrico, Cristiane Ribeiro Salmon, Walter Luiz Siqueira

**Affiliations:** 1 Department of Morphology, Genetics, Orthodontics and Pediatric Dentistry, School of Dentistry at Araraquara, UNESP-São Paulo State University, Araraquara 14801-903, SP, Brazil; francois.isnaldo@unesp.br (F.I.D.C.); samiactcorbi@yahoo.com.br (S.C.T.C.); diego.bussaneli@unesp.br (D.G.B.); 2 Department of Diagnosis and Surgery, School of Dentistry at Araraquara, UNESP-São Paulo State University, Araraquara 14801-385, SP, Brazil; allinyb@yahoo.com.br (A.d.S.B.); silvana.orrico@unesp.br (S.R.P.O.); 3 Advanced Research Center in Medicine, Union of the Colleges of the Great Lakes (UNILAGO), São José do Rio Preto 15030-070, SP, Brazil; 4 Dental Research Division, School of Dentistry, Paulista University, São Paulo 04026-002, SP, Brazil; cris.salmon.bb@gmail.com; 5 College of Dentistry, University of Saskatchewan, Saskatoon, SK S7N 5E4, Canada; walter.siqueira@usask.ca

**Keywords:** periodontitis, diabetes mellitus, dyslipidemia, saliva, proteome

## Abstract

Human whole saliva contains informative proteins related to disease processes and is important for oral cavity homeostasis. The aim of this study was to investigate the global salivary proteomic profile, including functional enrichment analysis of individuals affected by combinations of poorly/well-controlled type 2 diabetes mellitus (T2DM), dyslipidemia (DL), and periodontitis (P) for the identification of potential disease biomarkers. Biochemical and periodontal evaluations were performed on 150 subjects divided into five groups according to disease combinations. Unstimulated saliva was collected, and proteomic analysis was performed using Liquid Chromatography Electrospray Ionization Tandem Mass Spectrometry (LC-ESI-MS/MS). Results were obtained by searching for the Homo sapiens database of the UniProt catalog using Proteome Discoverer 1.3. PANTHER GO-Slim (PANTHER version 19.0), and Cytoscape 3.10.1 (extensions Metascape) were used to access biological pathways. A total of 1762 proteins were identified in saliva samples. The proteomic profile of T2DM-DL-P groups (G1—poorly controlled T2DM; G2—well-controlled T2DM) were the most diverse and functionally enriched. In G1 and G3, the most abundant protein was TTN, with ENO-1 being highly enriched and associated with the aerobic glycolysis pathway and PFN1 associated with a pro-inflammatory environment. PFN1 was highly enriched in (G1) AKIRIN-2 (immune response), and in (G2) KMT2A (epigenetic regulation) and CALML3 (DNA metabolism and repair). For (G4), the highest abundant protein was RIMS-1, with VIRMA (cytoskeletal organization) being the most enriched. The global salivary proteomics analyses demonstrated significantly altered protein profiles in each group of different pathological combinations, providing new insights into their biology and identifying potential diagnostic and therapeutic candidates for these diseases of growing global concern.

## 1. Introduction

Saliva is one of the most complex, versatile, and important body fluids, supplying a wide range of physiological needs [1]. Saliva does not clot like blood, is cheaper to store and ship, does not require specialized training, and has been established as an excellent fluid for the detection of disease biomarkers due to its protein content, which is instrumental for understanding disease processes [2]. Saliva may be considered a mirror of oral and systemic health, as a result of its exchange with substances that compose the plasma liquid through active carriage, passive diffusion, or ultrafiltration by the membrane of a thin layer of epithelial cells separating the salivary ducts from the systemic circulation [3]. Approximately 20–30% of total salivary proteins are also present in plasma, and proteins in both fluids show comparable functional diversity and disease associations [4]. This evidence expands the potential for the use of whole saliva in health monitoring and the diagnosis of a variety of diseases, including type 2 diabetes mellitus (T2DM) [5,6], dyslipidemia (DL) [7,8], and periodontitis (P) [9].

The co-occurrence of T2DM, DL, and P is increasingly prevalent [10,11,12]. Growing evidence demonstrates that these diseases are intrinsically biologically related, as they are chronic and multifactorial conditions modulated by genetic, environmental, and behavioral factors [13,14]. Although P has the development of a pathogenic dysbiotic biofilm as its main etiological factor, T2DM and DL result predominantly from metabolic perturbations [15,16]. T2DM is characterized by chronic hyperglycemia as a consequence of non-autoimmune progressive defects in insulin secretion and/or its action, frequently related to insulin resistance [17]. DL results from increased levels of lipoproteins in the blood [15], and P is a consequence of oral supportive tissue destruction by subgingival periodontopathogenic bacteria, which stimulates an inflammatory cascade and immunological reactions in the host [16].

Another biological interconnection for the co-occurrence of T2DM, DL, and P is the presence of circulating low-grade pro-inflammatory mediators and increased production of reactive oxygen species (ROS) [18]. Higher cytokine levels accelerate lipid and adipose tissue mobilization from the liver, which elevates low-density lipoprotein (LDL) binding to endothelium and smooth muscle cells and LDL receptor gene transcription [19]. Therefore, metabolic dysregulation, caused by hyperglycemia and DL, shares common chronic inflammatory mechanisms with subgingival bacterial infection. This shared pathology ultimately induces the inflammatory progression of P [18,20].

The bidirectional relationship between P and diabetes mellitus has been largely investigated, and close collaboration among dentists and diabetologists is required and strongly recommended [21]. A systematic review of 53 observational studies confirmed a bidirectional relationship between P and diabetes mellitus. Notably, the prevalence of T2DM is significantly elevated in individuals with P compared to healthy individuals (OR = 4.04, *p* < 0.0001, adjusted for other risk factors) [22]. Furthermore, T2DM is estimated to increase the risk of developing P by up to 34% [23].

It is essential to explain the specificities in each pathological panel and the biological interrelationship between T2DM, DL, and P. Regarding the identification of salivary biomarkers for diagnosis and clinical management of these diseases combined, there have only been two previous studies evaluating the simultaneous occurrence of T2DM and P; however, no study has evaluated the presence of these three comorbidities. Therefore, the aim of the present study was to investigate the global salivary proteomic profile, including functional enrichment analyses, in individuals affected by a combination of poorly/well-controlled T2DM, DL, and P, to expand and deepen the knowledge of the interrelationship between these complex diseases.

## 2. Results

A total of 150 participants, comprising a similar distribution of males and females among the groups (*p* = 0.931), were recruited; their demographic, biochemical, physical, and periodontal characteristics are presented in Supplementary Table S1. Poorly controlled T2DM participants presented the highest fasting glucose, glycated hemoglobin (HbA1c), and insulin resistance levels, along with the most severe periodontal destruction (Supplementary Table S1). Well-controlled T2DM individuals showed improved glycemic control but displayed similar lipid abnormalities compared to the dyslipidemic groups. Periodontal parameters followed the gradient of metabolic impairment, confirming the biological link between systemic metabolic dysregulation and periodontal inflammation.

**Figure 1:**
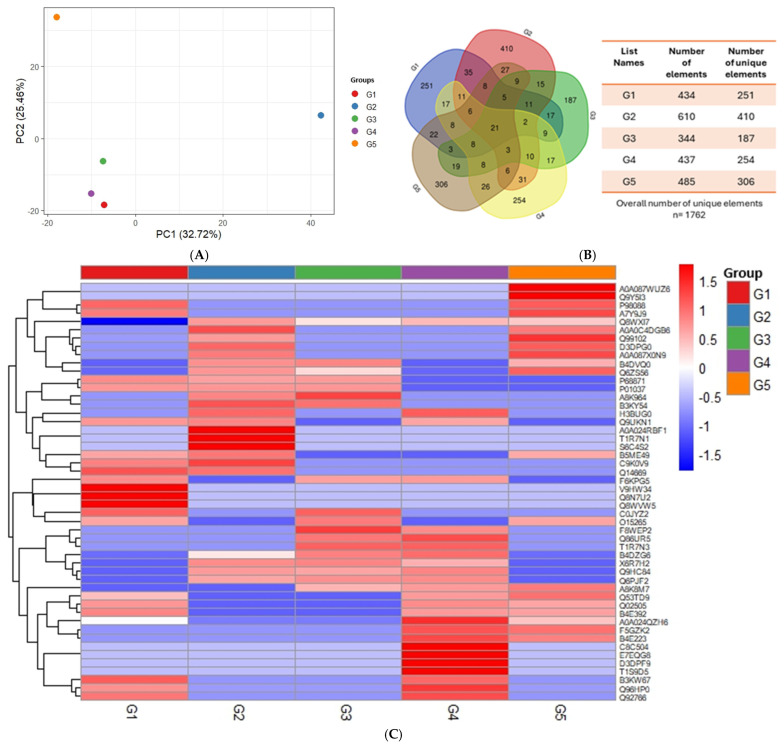
Proteomic profile of T2DMpoorly-DL-P (G1), T2DMwell-DL-P (G2), DL-P (G3), periodontitis (G4), and control (G5) groups (**A**) Principal component analysis (PCA) plot showing the differences between the samples of the G1, G2, G3, G4, and G5 groups. (**B**) Venn diagram showing the distribution of the total proteins identified in each group. (**C**) Heatmap illustrating the expression of the top 50 differentially abundant proteins in each group. Proteins are organized by descending fold-change (FC) values, which were colored by a directionality threshold of ≥1.5-FC (red = upregulated; blue = downregulated).

Mass spectrometry identified a total of 1762 salivary proteins. Principal component analysis revealed clear clustering between healthy controls, T2DMwell-DL-P, and groups with periodontitis, demonstrating that distinct metabolic and inflammatory conditions produce unique salivary proteomic patterns. The hierarchical clustering of the 50 proteins with the greatest differential abundance revealed well-defined proteomic patterns across all groups (Figure 1A–C). Distinct branches of the dendrogram separated T2DMpoorly-DL-P (G1), T2DMwell-DL-P (G2), DL-P (G3), periodontitis (G4), and control (G5), reflecting expression signatures specific to each group. Several protein clusters displayed consistent upregulation or downregulation within individual groups—for example, G1 showed a predominance of upregulated proteins compared with the other groups. These patterns indicate that each clinical condition is associated with a characteristic proteomic profile. Based on the list of proteins and the respective PSM values of each group, statistical analyses were applied to identify differentially expressed proteins. Results are shown in Supplementary Table S2, which ranked the 25 most upregulated proteins (with the highest Log2FC values) and the 25 most downregulated proteins (with the lowest Log2FC values) for each group comparison (Supplementary Table S3).

**Figure 2:**
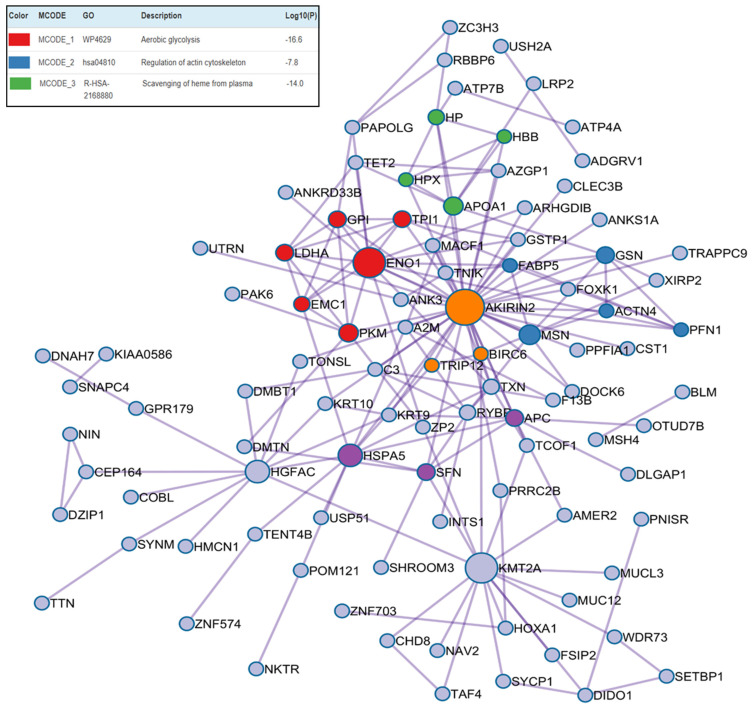
Protein–protein interaction enrichment analysis of upregulated differentially abundant proteins from the T2DMpoorly-DL-P versus control group comparison.

Distinct molecular signatures were observed for each disease combination. In T2DMpoorly-DL-P, proteins related to glycolysis, immune activation, and cytoskeletal organization were predominant, indicating a strong metabolic and inflammatory stress. Additionally, five main clusters were observed (each with a distinct color) in this protein–protein interaction. The main nodes were AKIRIN-2 (Akirin-2, orange node), ENO-1 (Enolase-1, red node), APOA-1 (Apolipoprotein A-I, green node), PFN-1 (Profilin-1, blue node), HBB (Hemoglobin subunit beta), and HSPA5 (Heat Shock Protein Family A Hsp70 member 5, purple node) with their respective seeds (Figure 2). Further details on the base-peak chromatogram for each group and the associated biological pathways and processes can be found in the Supplementary Figures S1 and S2.

The PPI network was formed using STRING and visualized with Cytoscape. Each node of the PPI structure represents a protein; edges between nodes demonstrate how they interact with each other. Network plots (clusters) were colored according to the MCODE-enriched term written in the same node color. Pathway and enrichment analyses were performed independently for each MCODE component, and the top three terms with the lowest *p*-values were presented with the functional descriptions of the corresponding components.

**Figure 3:**
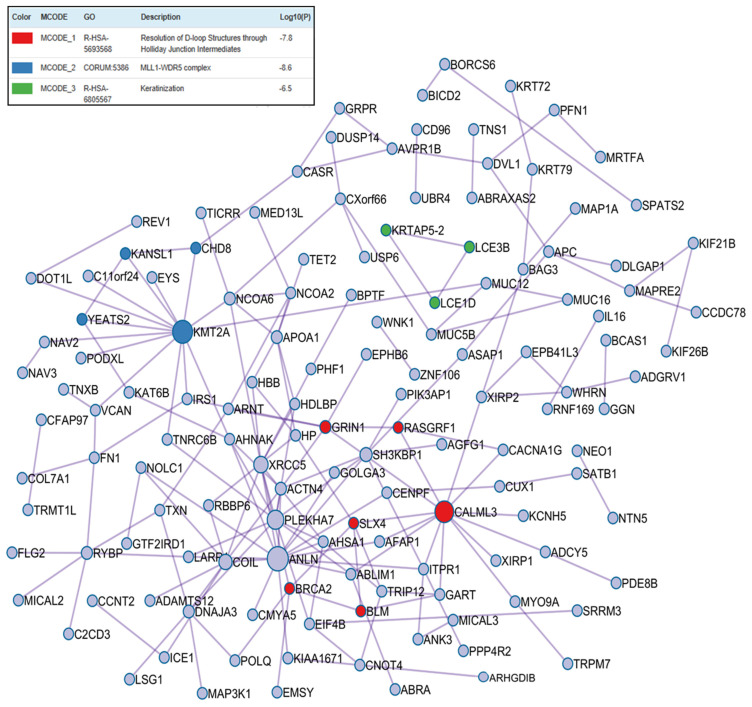
Protein–protein interaction enrichment analysis of upregulated differentially abundant proteins from the T2DMwell-DL-P versus control group comparison.

The T2MDwell-DL-P group exhibited enrichment in proteins associated with DNA repair, epigenetic modulation, and calcium signaling, which is consistent with adaptive and reparative processes resulting from glycemic stabilization. Figure 3 represented three enriched clusters for this group, with KMT2A (Lysine Methyltransferase 2A) and CALML3 (Calmodulin-like 3) hub proteins showing the most significant PPI network. Further details on the biological pathways and processes associated with this group can be found in Supplementary Figure S2.

**Figure 4:**
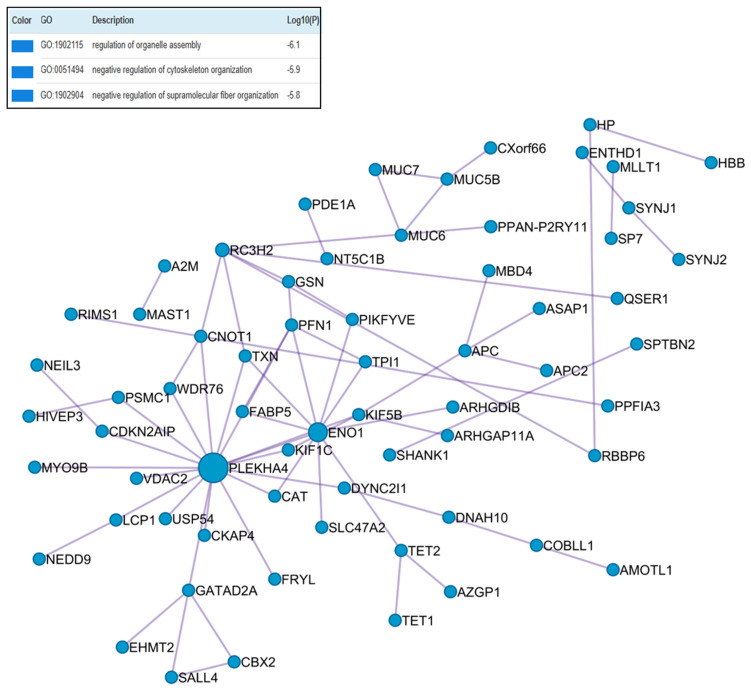
Protein–protein interaction enrichment analysis of upregulated differentially abundant proteins from the DL-P versus control group.

The DL-P group displayed moderate enrichment of cytoskeletal and organelle assembly proteins, suggesting lipid-driven alterations in cell structure and signaling. Figure 4 showed the PLEKHA4 (Pleckstrin homology domain-containing family A member 4) as the largest blue node protein, and this protein was exclusively found in DL-P group. ENO-1 and PFN-1 proteins also appeared as blue nodes, as they were enriched.

Pathway and process enrichment analyses were performed independently for each MCODE component, and the top three terms with the lowest *p*-values were presented with the functional descriptions of the corresponding components.

**Figure 5:**
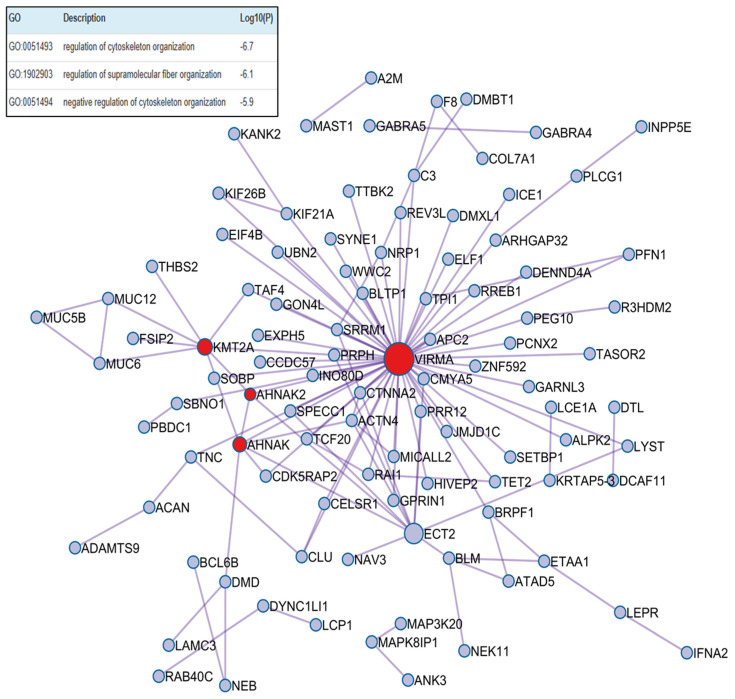
Protein–protein interaction enrichment analysis of upregulated differentially abundant proteins from the periodontitis versus control group.

The periodontitis-only group exhibited increased expression of proteins involved in cytoskeletal remodeling and cell junction regulation (with only one red MCODE cluster that physically connected the VIRMA (Vir-Like M6A Methyltransferase-Associated), AHNAK2, and KMT2A proteins), reflecting local inflammatory remodeling of periodontal tissues (Figure 5). Further details on the biological pathways and processes associated with this group can be found in Supplementary Figure S2.

**Figure 6:**
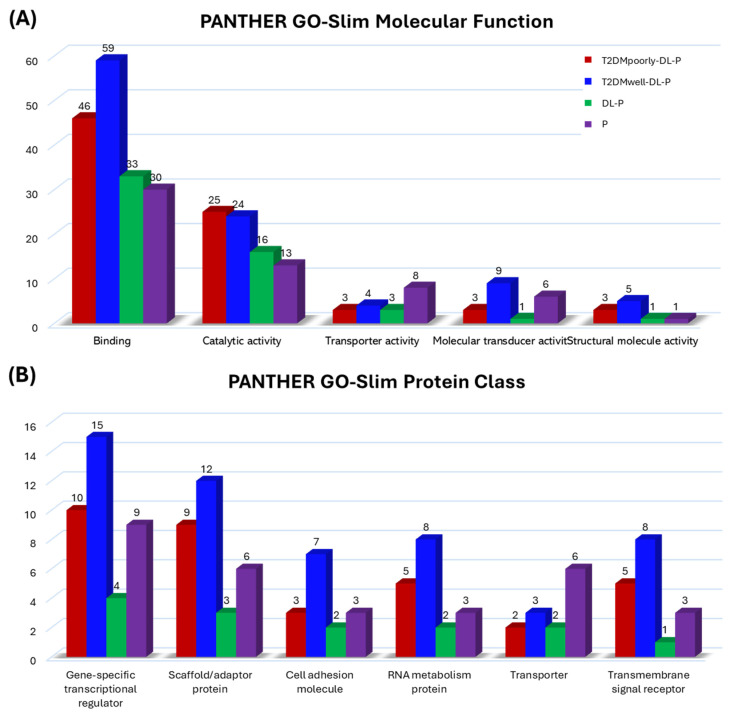
Enrichment analysis (molecular function) of upregulated differentially abundant proteins in each comparison between a diseased group and the control (PANTHER GO-Slim). (**A**) PANTHER GO-Slim Molecular Function, (**B**) PANTHER GO-Slim Protein Class.

Functional enrichment analysis supported these observations. T2DMpoorly-DL-P saliva was dominated by pathways associated with glycolysis, oxidative stress, and inflammation; the T2DMwell-DL-P group displayed enhanced molecular networks related to chromatin modification and cellular repair; and the DL-P group showed activation of membrane and cytoskeletal signaling processes. These findings demonstrated that systemic metabolic balance influenced circulating markers and the local salivary proteome, reflecting the interplay between systemic and oral inflammation (Figure 6). More details on the proteins identified in each category can be found in Supplementary Materials S2 and S3.

## 3. Discussion

To our knowledge, this is the first global salivary proteome profile of patients presenting combinations of poorly/well-controlled T2DM, DL, and P. These three diseases are of global concern, affecting thousands of patients worldwide. The data highlights the remarkable sensitivity of saliva to systemic metabolic regulation, emphasizing its potential as a noninvasive biomarker source for complex chronic diseases. As all the groups were formed with a similar distribution of males and females (*p* = 0.931), any aspect inherent to sex-based differences in salivary composition such as pH, protein, or lipid concentration did not affect the robustness of our hypothesis or analytical technique. Interestingly, the unsupervised method of PCA revealed a proteomic profile for T2DMwell-DL-P different from T2DMpoorly-DL-P as well as control, indicating that adequate metabolic control of T2DM impacts the proteomic profile of the groups. This agrees with the clinical observation that well-controlled T2DM subjects with P may receive similar periodontal treatment as individuals without T2DM [24]. Moreover, the similarity between the proteomic profile demonstrated by PCA and the heatmap for P and DL-P reinforces the biological connection between an oral pathology (P) and a systemic disease (DL), as demonstrated by meta-analyses [25].

A comprehensive literature review indicates that this is the first study to report significantly high levels of TTN (Titin) in the saliva of T2DM subjects (the highest in the T2DMpoorly-DL-P list). TTN (also known as Connectin) is the largest known human protein (∼4 MDa), constituting the so-called third filament component of muscle sarcomeres, and is a key component for sarcomere integrity, elasticity, and function [26]. As reviewed by Stroik et al. [27], T2DM patients have decreased phosphorylation of some amino acids in the N2B and PEVK regions, resulting in increased TTN-based stiffness. Improving insulin sensitivity to modulate TTN stiffness, through targeting neuroregulin-1 or via treatment with metformin, reverses the altered phosphorylation of the N2B and PEVK domains found in diabetes patients, showing its therapeutic potential for diastolic dysfunction [28]. In T2DM subjects, urinary TTN was detected as a biomarker of sarcopenia [29]. Similarly, in mouse models, the increase in urinary TTN concentration was associated with initial muscle damage and the onset of proteolysis, rather than with late-stage muscle wasting. Therefore, given that urinary TTN is a promising biomarker for detection of the onset of skeletal muscle catabolism and prediction of the subsequent development of muscle degeneration, noninvasive measurement of TTN may allow earlier detection of skeletal muscle proteolysis compared with conventional techniques. Salivary TTN detection, rather than urinary, is less invasive and more convenient to collect.

Also upregulated in T2DMpoorly-DL-P are AKIRIN-2 and ENO-1 which are involved, respectively, in adaptive immunity by promoting B-cell activation and stimulating immunoglobulin production [30]. As reviewed by Bosch et al. [30], growing evidence has demonstrated important roles of AKIRIN-2 in immune responses and tumorigenesis. Our finding of AKIRIN-2 association with diabetes is only the second to be reported in the literature. Previously, AKIRIN2 gene expression was found to be higher in the tubules of individuals with diabetic kidney disease than in that of healthy individuals [31]. ENO-1, a glycolytic enzyme that catalyzes the conversion of 2-phosphoglycerate to phosphoenolpyruvate [32], is also involved in the intravascular and pericellular fibrinolytic system due to its ability to serve as a receptor and activator of plasminogen on the cell surface of several cell-types such as leukocytes and neurons [33]. This protein, which was also found to be differentially expressed in DL-P individuals, is discussed in more detail below.

Of note was that the T2DMwell-DL-P subjects expressed a high number of upregulated proteins linked to many MF and PC terms, suggesting that glycemic control positively influences many aspects of metabolism. One of the positive effects of compensated T2DM is the enriched KMT2A protein (previously named MLL1) which is essential in epigenetic regulation. An important role for KMT2A is regulating macrophage-mediated inflammation in mouse wound repair, demonstrating a potential target for treatment of chronic inflammation in diabetic wounds [34]. In agreement with the positive metabolic aspect of glycemic control, we also observed CALML3 enriched for “calcium ion binding”, and “resolution of D-loop structures” together with BRCA2, BLM, SLX4. Interestingly, Metascape for the first time demonstrated a physical interconnection between CALML3 and DNA repair proteins (BRCA2—Breast Cancer Gene 2, BLM—Bloom syndrome protein, SLX4—Structure-Specific Endonuclease Subunit). While CALML3 itself has not been directly linked to D-loop resolution, calcium ions (Ca2+) are known to play a role in DNA repair, particularly in homologous recombination. Despite the few data regarding CALML3, it was reported that metformin suppresses gastric cancer through stimulating CALML3 secretion from gastric tumor--associated fibroblasts, representing a novel anti-cancer mechanism of metformin, and a tumor-suppressive role of CALML3 [35]. It is possible that the metformin utilized in treatment of T2DMwell-DL-P subjects might influence the upregulation of CALML3 protein, though the effects and mechanism underlying that protein warrant further investigation.

The PLEKHA4 protein found exclusively in DL-P subjects has lipid kinase activity [36] and binds specifically to phosphatidylinositol 3-phosphate but not to other phosphoinositides [37]. It appeared highly enriched in the network, described by GO as “regulation of organelle assembly”, because it acts as a signaling adaptor at the plasma membrane that plays a crucial role in regulating organelle assembly, particularly by modulating Wnt and planar cell polarity (PCP) signaling. PFN-1 and ENO-1 were enriched in the PPI of subjects that have both DL and P. Furukawa et al. [38] also found that in the salivary proteome, PFN-1 in the T2DM+P group and ENO-1 in individuals with P were upregulated compared with the healthy group. Since subjects enrolled in the Furukawa et al. [38] study were not evaluated for DL, there is the potential that DL could be present in those subjects.

The disease-related roles of ENO-1 are mostly attributed to its immunogenic capacity, DNA-binding ability, and plasmin(ogen) receptor function, which are significantly affected by its three-dimensional structure and surface properties, rather than its enzymatic activity [39]. Our findings show actin-binding protein PFN-1 enriched in the saliva of individuals with DL-P and T2DMpoorly-DL-P, consistent with observations [38] in T2DM+P patients. These results add to the evidence demonstrated by a meta-analysis [9] where 4 of 13 salivary proteomic studies reported PFN-1 upregulated in P subjects [40,41,42,43].

Clinical evidence agreed with our observation of the enriched association of PFN-1 with DL, showing its enhanced expression in atherosclerotic plaques, with a positive correlation to a pro-inflammatory environment and significant macrophage infiltration [44,45]. PFN-1 was identified as part of the HDL proteome [46], and after treatment with rosuvastatin there was decreased PFN-1 expression, with these levels being mostly dependent on the subjects’ inflammatory phenotype [47]. These findings contribute to the hypothesis of PFN-1 as a biomarker for atherogenesis, diabetes, and myocardial infarction [48].

The most abundant protein found in individuals with P was RIMS1 (Regulating Synaptic Membrane Exocytosis 1) that regulates synaptic vesicle exocytosis [49], but as this is the first study to report it, a discussion of previous research is not possible. The highest enriched protein in P group was VIRMA (Vir-Like M6A Methyltransferase-Associated), which is a component of a complex that mediates N6-methyladenosine (m6A) methylation of RNAs, a modification that plays a role in the efficiency of mRNA splicing and RNA processing [50]. AHNAK (AHNAK Nucleoprotein) may play a role in diverse processes such as blood–brain barrier formation, cell structure and migration, cardiac calcium channel regulation, and tumor metastasis [51]. Alam et al. [52] completed a robust bioinformatics analysis and found AHNAK as a hub gene with potential to be used as a biomarker of T2DM, obesity, P, and oral cancer. The AHNAK2 (AHNAK n-2) protein may play a role in calcium signaling by associating with calcium channel proteins. Diseases associated with AHNAK2 include Charcot–Marie–Tooth (CMT) disease type 4F, and according to GWAS studies, systemic lupus erythematosus, rheumatoid arthritis, and systemic scleroderma [53]. CMT disease, a progressive neurological disorder affecting peripheral nerves [53], can lead to weakness and motor impairments, hindering individuals from brushing and cleaning their teeth effectively, increasing the risk of P. However, P could be not only a consequence of CMT disease but a comorbidity, whose common biological mechanism is not yet known. Increasing evidence demonstrates comorbidity between P and neurodegenerative diseases (ND) such as CMT and Alzheimer’s disease; potentially because P contributes to systemic inflammation, influencing the development of ND [54].

The presence of LYST (Lysosomal Trafficking Regulator) protein was noted in the PPI of P group, which is associated with cognitive impairment and Chediak–Higashi Syndrome (CHS), a condition that historically exhibits aggressive P. LYST mutations in CHS patients may affect TLR-2 and TLR-4 expression and function, leading to dysregulated immunoinflammatory response, which in turn may influence the periodontal phenotype [55]. LCP1 (Lymphocyte Cytosolic Protein 1) and PIP (Prolactin Induced Protein) were also observed in the PPI of P group. Both were expressed in all five groups but found primarily in P group (Supplementary Table S2). Among the related pathways of LCP1 are cytokine signaling in immune system, calcium ion binding, and actin binding. The actin-binding function is identical to the function reported for PIP.

One of the main limitations of our study is that we utilized a pooled sample for each group, and we are not able to develop another methodology to assess the presence or quantity of a specific protein in the subjects’ groups, as we did not have extra salivary aliquots. When the proteome is analyzed in a pooled sample composed of all participants in a group, the possibility of monitoring individual variability is lost. Ventura et al. [56] compared proteome analysis of salivary samples pooled from all 10 volunteers in a group, with proteome analysis of an individual sample from one selected volunteer, obtaining very similar proteome results. Despite these limitations and considering that the aim of this study was to present the global salivary proteomic analysis associated with T2DM, DL, and P, we achieved this goal with high confidence, producing several novel findings that are in agreement with the literature. Moreover, recognizing that the saliva collection method heavily impacts the individual proteomic profile, and that the lack of standardized collection and handling procedures among different studies challenges data comparison, we employed a detailed and standardized method for saliva collection and processing. Our results may lead other researchers to perform further studies with a higher number of subjects and ethnically diverse subjects to replicate our findings, and strictly utilizing the same clinical criteria and analytical methods presented here is essential.

## 4. Materials and Methods

### 4.1. Study Population and Clinical Evaluations

Subjects of similar demographic background, who have never smoked, aged from 35 to 60 years, and who have at least 15 natural teeth were enrolled in this study after being informed about the aims and methods and after providing their written consent to participate in this study. This study was approved by the Ethics in Human Research Committee of School of Dentistry at Araraquara (UNESP—São Paulo State University, Araraquara, Brazil; Protocol number 50/06) and was conducted according to the ethical principles of the Declaration of Helsinki. The selected subjects had their glycemic and lipid profiles investigated by biochemical blood analysis (after a 12-h overnight fast) and were submitted to full periodontal examination.

Clinical inclusion criteria included the following: (i) T2DM subjects that were diagnosed by an endocrinologist and who monitored their glycemic control by evaluation of HbA1c were divided into poorly controlled subjects (HbA1c ≥ 8.5%, 64 mmol/mol) or well-controlled subjects (HbA1c < 7.0%) [57]. Subjects were considered nondiabetic (normoglycemic individuals) if they presented fasting glucose levels < 100 mg/dL and HbA1c < 5.7%; (ii) to avoid the inclusion of individuals with transitory dyslipidemia (DL), the cutoff values were the highest according to the National Cholesterol Educational Program (NCEP) Adult Treatment III (ATP III): total cholesterol (TC) ≥ 240 mg/dL, low-density lipoprotein (LDL) ≥ 160 mg/dL, high density lipoprotein (HDL) ≤ 40 mg/dL, and triglycerides (TG) ≥ 200 mg/dL [58]; (iii) all patients were subjected to a full-mouth periodontal examination performed at six sites per tooth by a single trained calibrated examiner (A.B.S., Kappa = 0.89). Periodontal pocket depth, clinical attachment loss, and bleeding on probing were evaluated with a periodontal probe PCP UNC15-6 (Hu-Friedy^®^, Chicago, IL, USA), as previously described [57]. Diagnosis of P as defined by the American Academy of Periodontology, classified individuals in stages III and IV of P as well as in grades A, B, and C [59].

The 150 subjects in this study were divided into five groups (G) of 30 subjects each, based on diabetic, dyslipidemic, and periodontal status: poorly controlled T2DM with DL and P (G1), well-controlled T2DM with DL and P (G2), normoglycemic individuals with DL and P (G3), normoglycemic individuals with P (G4), and a control group of systemically and periodontally healthy individuals (G5).

### 4.2. Whole Saliva Sample Collection and Preparation

Subjects were advised to fast from food and drink and not to perform toothbrushing in the early morning before undergoing blood exams. Following the completion of blood collection for biochemical analyses in the fasted state between 8:00 a.m. and 9:00 a.m, all participants were immediately transferred to the Dental Clinic for saliva sampling. Saliva was collected between 8:30 a.m. and 10:00 a.m. Each subject was comfortably seated and received an individual sterile Falcon tube that was kept on ice throughout the collection procedure. Participants collected non-stimulated saliva using the resting method for a minimum duration of 5 min. The minimum required whole saliva volume was 2 mL, collected within a maximum time of 10 min. The maximum whole saliva volume collected per participant was 5 mL. After collection, each saliva sample was centrifuged at 14,000× *g* for 20 min at 4 °C, and the supernatant was divided into 1.5 mL aliquots which were frozen at −80 °C. Salivary samples were prepared according to a previous study [56] and had their total protein concentration measured by the bicinchoninic acid assay (Pierce Chemical, Rockford, IL, USA) using BSA as a protein standard.

### 4.3. In–Solution Digestion

An equal 20 μg protein amount from each subject within the same group was combined to constitute a pooled sample representative of that group [38,56,60,61]. These samples were dried in a rotary evaporator, denatured, and reduced for 2 h by the addition of 50 μL of 4 M urea, 10 mM dithiothreitol (DTT), and 50 mM NH_4_HCO_3_, pH 7.8, and incubated for 1 h at room temperature. The solution was diluted with the addition of 150 μL of 50 mM NH_4_HCO_3_, pH 7.8. After the addition of 2% w/w sequencing-grade trypsin (Promega, Madison, WI, USA), tryptic digestion was carried out for a minimum of 16 h at 37 °C in a water bath. Finally, samples were dried in a rotary evaporator, de-salted by C-18 ZipTip1Pipette Tips (Millipore, Billerica, MA, USA), and subjected to mass spectrometry [62].

### 4.4. Liquid Chromatography Electrospray Ionization Tandem Mass Spectrometry (LC-ESI-MS/MS)

After trypsinization, an equal amount of all the samples was resuspended in 15 μL of 97.5% H_2_O/2.4% acetonitrile/0.1% formic acid and then subjected to reversed-phase LC-ESI-MS/MS, using an LTQ-Velos (Thermo Scientific, San Jose, CA, USA) mass spectrometer. Liquid chromatography separation was achieved using a C18 column of capillary fused silica (column length 10 mm, column ID 75 μm, 3 μm spherical beads, and 100 Å pore size) linked to the mass spectrometer through electrospray ionization. Peptides were eluted from the nanoflow reversed-phase HPLC over linear 85 min gradient ranging from 5% to 55% of solvent B (97.5% acetonitrile, 0.1% formic acid), at a flow rate of 300 nL/min, with a maximum pressure of 280 bar. Electrospray voltage and the temperature of the ion-transfer capillary were 1.8 kV and 250 °C, respectively. The survey scan was set in the range of *m*/*z* values 390–2000 MS/MS. Each survey scan (MS) was followed by an automated sequential selection of seven peptides for a standard collision-induced dissociation (CID), with dynamic exclusion of the previously selected ions [62].

### 4.5. Protein Identification

The acquired MS/MS spectra were searched against human protein databases (Swiss PROT and TrEMBL, Swiss Institute of Bioinformatics, Geneva, Switzerland, http://ca.expasy.org/sprot/, accessed on 15 October 2016) using Proteome Discoverer 1.3 software (Thermo Scientific, San Jose, CA, USA) and SEQUEST algorithm. The search parameters for SEQUEST included 1.5; 2.5; 3.1; 3.1; 4.5. Parameter Xcorr was used. A filter was applied to the peptide sequences during the import that eliminated all sequences with a Percolator q-value greater than 1% (false discovery rate). Peptides were grouped into proteins, and a protein ratio and *p*-value were calculated [62]. A total of 5 runs per group were carried out in the mass spectrometer. For positive identification, the same protein had to be identified in at least three runs from the same group.

### 4.6. Bioinformatics and Statistical Analysis

Resulting quantitative values (spectrum counts, PSM) were used to analyze the distribution of identified proteins in each group of subjects. Only proteins identified with at least two unique peptides were considered in the results. The principal component analysis (PCA) of each studied group was performed on raw values converted to the log2 scale. The R statistical software function “prcomp” then determined the principal component values, with graphical representation provided by the “ggfortify” package (from ‘ggplot2′). The heatmap organization was constructed by converting NSC into z-scores and submitting them to the “pheatmap” package. Proteins identified in each group were compared using the Venny 2.0 online tool, an interactive tool for comparing lists with Venn’s diagrams (http://bioinfogp.cnb.csic.es/tools/venny/index.html) accessed on 10 April 2025 [63]. For enrichment analysis of upregulated differentially expressed proteins in each group comparison, we utilized Cytoscape (3.10.1) and its extensions Metascape and PANTHER GO-Slim (PANTHER version 19.0), (http://www.pantherdb.org/, accessed on 6 April 2025). Proteins identified in each group were compared using the Venny 2.0 online tool. Fold-change values (FC ≥ ±10.0 and FC ≤ −10.0) of each diseased (G1, G2, G3, G4) group compared with the control (G5) group were determined (i.e., G1 versus G5; G2 versus G5; G3 versus G5; G4 versus G5) to obtain lists of up/downregulated proteins (Log2 Fold Change, Log2FC) as well as to perform enrichment analyses.

Pathway and enrichment analyses of upregulated differentially abundant proteins of each group comparison were carried out using the Metascape.org platform [64] with the following ontology sources: Gene ontology (GO) Biological Processes, PANTHER Pathway, KEGG Pathway, Reactome Gene Sets, WikiPathways, Canonical Pathways, and CORUM. All genes in the genome were used as an enrichment background. Terms with a *p*-value < 0.01, a minimum count of 3, and an enrichment factor > 1.5 were collected and grouped into clusters based on their membership similarities. The enrichment factor is the ratio between the observed counts and the counts expected by chance. *p*-values were calculated based on the cumulative hypergeometric distribution, and q-values were calculated using the Benjamini–Hochberg procedure to account for multiple testing.

From Metascape, the upregulated differentially abundant proteins of each group comparison were submitted to Cytoscape (version 3.10.1, Cytoscape Consortium, USA) [65] to visualize the protein network plot. The circle node color is paired with the enriched terms that represent cluster identity (text written in the same color in the figure) and node size is proportional to the number of input genes included in the selected terms. Terms with a Kappa similarity score > 0.3 are linked by a branch whose thickness represents the similarity score [64]. To assess the enriched physical protein–protein interactions (PPI), the BioGrid, InWeb_IM, OmniPath, and STRING (Search Tool for the Retrieval of Interacting Genes/Proteins) databases were used. Only physical interactions from STRING (with a physical score > 0.132) and BioGrid were considered. The resulting networks include only proteins that form physical interactions with at least one other protein in the list. When the network contained between 3 and 500 proteins, the MCODE (Molecular Complex Detection, https://apps.cytoscape.org/apps/mcode, accessed on 6 April 2025) algorithm feature of the Cytoscape tool was applied to identify densely connected network components. Pathway and enrichment analyses were performed independently for each MCODE component, and the top three terms with the lowest *p*-values were presented with the functional description of the corresponding components. Through MCODE clustering, extremely interrelated regions are identified, revealing the substantial components inside the PPIs structure [66].

The PANTHER GO-Slim (PANTHER version 19.0), accessed on 6 April 2025 (http://www.pantherdb.org/), was utilized for the enrichment analysis of differentially abundant proteins in each group comparison, analyzed according to Molecular Function (MF) and Protein Class (PC) terms. To enable qualitative comparison among groups, the number and abundance of proteins participating in specific pathways within each comparative group were quantitatively represented by a bar graph, detailing the proteins identified in each enriched term.

The distribution and normality of the characteristics of each group were evaluated by the D’Agostino–Pearson test. The Chi-squared test (for sex) and Kruskal–Wallis’ test, followed by Dunn’s post-test were used to compare other sample characteristics by using GraphPad Prism software version 5.0. Differentially expressed proteins used for pathway enrichment analysis with Metascape were defined as those with *p* < 0.05 in this study. Proteome data was analyzed using R software (version R-4.5., RStudio extension 2025.05.1+513) and followed by Student’s *t*-test (*p* < 0.05). The fold-change was calculated as the ratio of G1, G2, G3, and G4/G5 values.

## 5. Conclusions

Saliva reflects the biological interplay between diabetes, dyslipidemia, and periodontitis. Poor glycemic control leads to a proteomic profile dominated by inflammatory and glycolytic activity, while adequate control restores regulatory and repair-associated pathways. Proteins such as Titin, Akirin-2, Enolase-1, and Calmodulin-like 3 emerge as promising biomarker candidates linking systemic metabolism to oral inflammation. These findings support the use of salivary proteomics as a powerful, noninvasive approach for identifying and monitoring metabolic–inflammatory disease interactions.

## Data Availability

The original contributions presented in this study are included in the article/Supplementary Materials. Further inquiries can be directed to the corresponding author.

## Supplementary Materials

The following supporting information can be downloaded at: https://www.mdpi.com/article/10.3390/ijms262411778/s1.

## Abbreviations

The following abbreviations are used in this manuscript:

T2DM	Type 2 Diabetes Mellitus
DL	Dyslipidemia
P	Periodontitis
LC-ESI-MS/MS	Liquid Chromatography Electrospray Ionization Tandem Mass Spectrometry
LDL	Low-Density Lipoprotein
OR	Odds Ratio
HbA1c	Glycated Hemoglobin
PSM	Spectrum Counts
ENO-1	Enolase-1
APOA-1	Apolipoprotein A-I
PFN-1	Profilin-1
HBB	Hemoglobin Subunit Beta
HSPA5	Heat Shock Protein Family A Hsp70 Member 5
KMT2A	Lysine Methyltransferase 2A
CALML3	Calmodulin-Like 3
PLEKHA4	Pleckstrin Homology Domain-Containing Family A Member 4
PCA	Principal Component Analysis
TTN	Titin
BRCA2	Breast Cancer Gene 2
BLM	Bloom Syndrome Protein
SLX4	Structure-Specific Endonuclease Subunit
RIMS-1	Regulating Synaptic Membrane Exocytosis 1
VIRMA	Vir-Like M6A Methyltransferase-Associated
AHNAK	AHNAK Nucleoprotein
LYST	Lysosomal Trafficking Regulator
CHS	Chediak–Higashi Syndrome
LCP1	Lymphocyte Cytosolic Protein 1
PIP	Prolactin-Induced Protein
NCEP	National Cholesterol Educational Program
ATP III	Adult Treatment III
TC	Total cholesterol
LDL	Low Density Lipoprotein
HDL	High Density Lipoprotein
TG	Triglycerides
STRING	Search Tool for the Retrieval of Interacting Genes/Proteins
MF	Molecular Function
PC	Protein Class
