# Supplementary material for: Global Salivary Proteomic Profiling of Individuals with the Co-Occurrence of Type 2 Diabetes Mellitus, Dyslipidemia, and Periodontitis
Source: Int J Mol Sci. 2025 Dec 5;26(24):11778. doi: 10.3390/ijms262411778 (PMC12732424; doi:10.3390/ijms262411778)
Supplement: Supplementary file 1 [file ijms-26-11778-s001.zip › Supplementary Materials S1.pdf]

Supplementary Material

Methods

The subjects were excluded based on the following criteria: history of antibiotic therapy in the previous 3 months and/or nonsteroidal anti-inflammatory drug therapy in the previous 6 months, pregnancy or use of contraceptives or any other hormone, current smokers or former smokers, history of anemia, periodontal treatment or surgery in the preceding 6 months, use of hypolipemic drugs such as statins or fibrates, and history of diseases that interfere with lipid metabolism, such as hypothyroidism and hypopituitarism. Subjects with T2DM were recruited from local outpatient diabetic clinics, regional community hospitals, and physicians' offices. Normoglycemic individuals were consecutively selected from the list of subjects who voluntarily sought dental treatment at the School of Dentistry at Araraquara (UNESP – São Paulo State University, Araraquara, Brazil), at the same time as the T2DM groups.

Biochemical, Physical and Periodontal evaluations

Blood samples were collected after a 12-hour overnight fast for the evaluation of fasting plasma glucose (mg/dL) by modified Bondar & Mead method, glycated haemoglobin (HbA1c) by enzymatic immunoturbidimetry, insulin levels by the chemiluminescence method (U/L), high-sensitivity C-reactive protein by the nephelomeric method. Insulin resistance was also evaluated for insulin levels for calculation of the homeostasis model assessment (HOMA-IR) of the insulin resistance index. Lipid profile – total cholesterol (TC), triglycerides (TGs), and HDL – were evaluated by enzymatic methods. LDL was determined by the Friedewald formula. The same laboratory performed all analyses. Each subject was submitted to physical examination including anthropometric data such as abdominal circumference (cm), hip (cm), waist (cm) and height (m), weight (kg), and body mass index (BMI).

Results

Supplementary Table S1 presents the group's comparisons of demographic, biochemical, physical and periodontal characteristics. Proteins found in common to all five groups and their biological/molecular functions are presented in Supplementary Table S2. Diabetic groups (T2DMpoorly-DL-P and T2DMwell-DL-P) exhibited similar insulin and HOMA insulin resistance levels. These groups were also obese and have the highest abdominal circumference of casuistic.

Supplementary Table S1. Demographic, biochemical, physical and periodontal characteristics of the subjects (n=30 each group)

Variable<br>Median (25th-75th IQR)	(G1)T2DMpoorly-DL-P<br>n= 30	(G2) T2DMwell-DL-P<br>n= 30	(G3)DL-P<br>n= 30	(G4)Perio<br>n= 30	(G5)Control<br>n= 30
#Gender (M/F)	12/18	10/20	13/17	11/19	12/18
Age	48.5 (41.2-55)[a]	52 (46.5-56)[a]	51 (43-55)[a]	45 (40.5-47)[a]	40.5 (36-42)
*BMI (Kg/m^2)	28.9 (26.8–34.4)[a,b]	30.5 (29.1–33.3)[a,b]	28.0 (26.1–30.9)	26.2 (23.7–29.3)	23.7 (22.2–27.5)
*Waist-to-hip ratio	1.0 (0.9–1.0) [a,b]	1.0 (0.9–1.0) [a,b]	0.9 (0.9–1.0)[a]	0.9 (0.9–1.0)[a]	0.8 (0.8–0.9)
*Abdominal circumference (cm)	101.5 (97.0–111.8)[a]	110.0 (102.0–117.0)[a,b,c]	98.0 (93.0–106.0)[a]	96.0 (92.0–105.0)	85.5 (79.8–97.5)
*Hs C-reactive protein (mg/dL)	0.2 (0.1–0.7)	0.5 (0.2–1.1)[a,b]	0.2 (0.1–0.5)	0.2 (0.0–0.4)	0.1 (0.0–0.3)
Fasting glucose (mg/dL)	209.5 (169.5–290.0)[a,b,c,d]	125.0 (111.0–162.0)[b,c]	89.0 (86.0–95.0)	91.0 (86.0–99.0)	86.0 (80.0–92.0)
Insulin (U/L)	13.1 (8.8–17.6)[a,b]	13.9 (9.8–19.9)[a,b]	11.0 (5.4–19.9)	7.7 (5.3–10.7)	6.5 (4.7–7.8)
HbA_{1c} (%)	9.7 (8.9–11.4)[a,b,c,d]	6.5 (6.2–7.4)[a,b,c]	5.5 (4.7–5.9)	5.1 (4.7–5.4)	5.4 (5.3–5.5)
HOMA-IR	6.4 (4.5–10.0)[a,b,c]	5.2 (3.0–7.6)[a,b,c]	2.2 (1.2–4.4)	1.6 (1.2–2.7)	1.3 (1.0–1.6)
Total cholesterol (mg/dL)	244.0 (225.3–266.3)[a,b]	253.0 (201.0–271.0)[a,b]	249.0 (220.0–264.0)[a,b]	179.0 (161.0–190.0)	181.5 (163.3–197.0)
HDL cholesterol (mg/dL)	41.5 (39.0–51.3)	43.0 (40.0–53.0)	49.0 (43.0–58.0)	47.0 (41.0–51.0)	48.5 (44.0–53.0)
Non-HDL-C	192.0 (177.3–220.5)	203.0 (161.0–222.0)	195.0 (172.0–210.0)	121.0 (110.0–142.0)	132.5 (115.0–144.3)
LDL cholesterol (mg/dL)	160.1 (130.3–174.3)[a,b]	151.0 (109.0–184.2)[a,b]	162.6 (134.8–180.2)[a,b]	102.2 (92.2–116.4)	113.9 (98.6–126.8)
Triglycerides (mg/dL)	219.0 (137.8–270.3)[a,b]	253.0 (206.0–308.0)[a,b]	200.0 (134.0–231.0)[a,b]	89.0 (65.0–126.0)	85.0 (64.3–105.3)
Percentage of sites with visible plaque	79.9 (68.2–88.3)[a,b]	72.1 (64.6–78.7)[a]	69.0 (62.0–79.8)[a]	53.8 (47.9–75.0)[a]	16.1 (9.8–20.0)
Percentage of sites with marginal bleeding	60.5 (48.1–70.2)[a,b,c]	45.0 (36.3–58.7)[a]	35.9 (30.0–46.6)[a]	40.2 (32.6–50.0)[a]	8.0 (5.4–11.5)
Percentage of sites with bleeding on probing	69.2 (58.3–80.7)[a,b,c,d]	56.7 (44.7–64.1)[a]	51.2 (44.0–59.1)[a]	53.8 (46.3–60.7)[a]	12.7 (10.0–16.8)
Number of teeth	22.0 (19.8–26.0)[a]	21.0 (18.0–26.0)[a]	24.0 (21.0–26.0)[a]	25.0 (22.0–27.0)[a]	28.0 (27.0–28.0)
Percentage of sites with probing depth ≤ 3 mm	46.0 (32.8–52.0)[a,b,c,d]	61.0 (47.0–66.0)[a]	65.0 (56.0–73.0)[a]	48.0 (44.0–60.0)[a]	99.0 (98.8–100.0)
Percentage of sites with probing depth = 4–5 mm	32.0 (22.8–38.0)[a,b]	30.0 (25.0–37.0)[a,b]	28.0 (24.0–39.0)[a,b]	42.0 (38.0–48.0)[a]	1.0 (0.0–1.3)
Percentage of sites with probing depth ≥ 6 mm	26.5 (8.8–37.3)[a,b,c,d]	11.0 (4.0–18.0)[a,c]	5.0 (2.0–9.0)[a]	4.0 (2.0–10.0)[a]	0.0 (0.0–0.0)
Percentage of sites with attachment loss ≤ 2 mm	12.5 (7.8–15.0)[a]	16.0 (4.0–20.0)[a]	24.0 (15.0–33.0)[a,b]	4.0 (1.0–17.0)[a]	63.5 (58.0–74.0)

Percentage of sites with attachment loss = 3–4 mm	37.5 (26.8–46.0)[b,c]	48.0 (37.0–57.0)[a,b]	52.0 (44.0–58.0)[a]	62.0 (55.0–69.0)[a,c]	36.5 (26.0–42.0)
Percentage of sites with attachment loss ≥ 5 mm	47.0 (40.5–56.3)[a,b, c, d]	31.0 (22.0–46.0)[a,c]	21.0 (15.0–30.0)[a]	29.0 (22.0–36.0)[a]	0.0 (0.0–0.0)
Number of sites with suppuration	5.5 (2.0–12.5)[a,b,c,d]	1.0 (0.0–4.0)[a]	1.0 (0.0–1.0)	0.0 (0.0–2.0)	0.0 (0.0–0.0)

IQR = interquartile range. # males/females' similar distribution among groups (p=0.931), Chi-squared test. *cardiovascular risk factors. Hs C-reactive protein = high sensitive C-reactive protein. [a]$p < 0.05$ in comparison to (G5)Control group; [b]$p < 0.05$ in comparison to (G4)Perio group; [c]$p < 0.05$ in comparison to (G3)DL-P (Dyslipidemia-Periodontitis) group; [d]$p < 0.05$ in comparison to (G2)T2DMwell-DL-P (well-controlled-Type 2 Diabetes Mellitus-Dyslipidemia-Periodontitis) group according to Kruskal-Wallis test, and Dunn's post-test.

Supplementary Table S2. Salivary proteins in common in all five groups and their molecular function. Quantitative protein values are PSM (spectrum counts).

UniProt Accession	Description	Protein Symbol	G1	G2	G3	G4	G5
**Cell organization**							
E7EVA0	Microtubule-associated protein	MAP4	19	12	28	34	26
P07737	Profilin-1	PFN1	14	13	13	12	5
A0A024RC30	Desmoglein 3 (Pemphigus vulgaris antigen), isoform CRA_a	DSG3	10	3	12	7	6
P13796	Plastin-2	LCP1	7	6	11	16	3
**Enzyme regulator**							
P01036	Cystatin-S	CST4	47	48	43	36	38
P04080	Cystatin-B	CSTB	8	10	9	7	9
**Immune response**							
P01833	Polymeric immunoglobulin receptor	PIGR	19	17	31	16	17
P12273	Prolactin-inducible protein	PIP	18	14	23	25	21
Q8IVL0	Neuron navigator 3	NAV3	8	30	5	30	7
**Metabolism**							
B7ZMD7	Alpha-amylase	AMY1A	102	82	130	107	105
G3CIG0	MUC19 variant 12	MUC19	81	144	81	63	81
A0A0C4DGN4	Zymogen granule protein 16 homolog B	ZG16B	17	13	15	17	24
P06733	Alpha-enolase	ENO1	15	7	12	8	4
A0A087WTM7	Apolipoprotein B-100	APOB	7	19	16	8	13
**Signal transduction**							
E9PAV3	Nascent polypeptide-associated complex subunit alpha, muscle-specific form	NACA	37	61	26	11	22
P27482	Calmodulin-like protein 3	CALML3	13	17	10	7	7
P63104	14-3-3 protein zeta/delta	YWHAZ	7	3	5	7	6
**Transport**							
B2R7Z6	cDNA, FLJ93674	B2R7Z6	6	5	5	5	3
P31025	Lipocalin-1	LCN1	5	5	7	5	7
**Non specified**							
P02814	Submaxillary gland androgen-regulated protein 3B	SMR3B	22	19	38	64	106
P23280	Carbonic anhydrase 6	CA6	19	12	20	18	18

G1: T2DMpoorly-DL-P; G2: T2DMwell-DL-P; G3: DL-P; G4: Perio; G5: Control

Salivary proteomic profile analyses

After protein elution and trypsinization, equal amount of peptides were subjected to nanoscale LC-ESI-MS/MS. A total of 5 runs per group were carried out. The base-peak chromatogram for reversed-phase chromatography monitored by the mass spectrometer represents the intensity of all peptide ions in the sample in a single scan. Salivary proteome from all five different groups showed a consistent elution of protein/peptides range from 10 to 60 min (Supplementary Figure S1). The Venn diagram showing a total of 1762 identified proteins, also demonstrated 21 (1.19%) proteins in common to all five groups (Supplementary Table S2).

The abundance variation in each comparison of a diseased group with the control is presented in the Supplementary Table S3, as a list of 25 up-regulated and 25 down-regulated proteins (Log2FC).

Supplementary Figure S1. Example of base-peak chromatogram of each group. Peptide separation was achieved using a nano-flow reverse-phase HPLC column, with gradient elution ranging from 5 to 55% solvent B in 85 min.

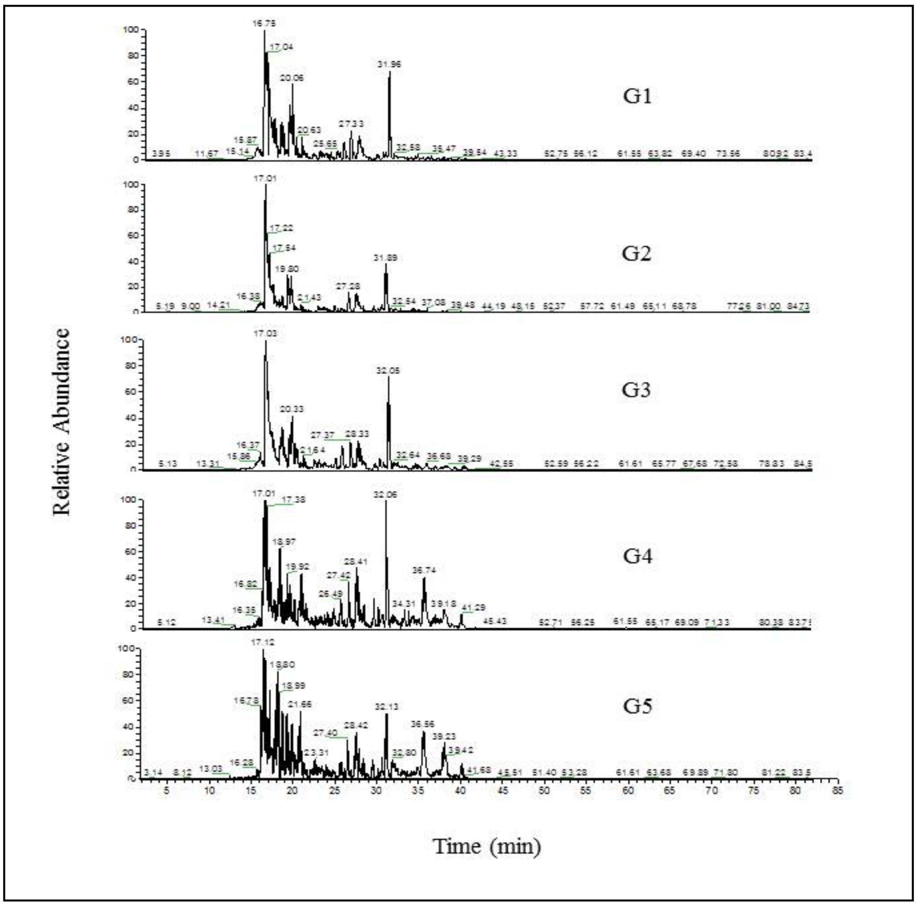

Supplementary Table S3. Abundance variation in each comparison of a diseased group with the control. 25 up-regulated and 25 down-regulated proteins in each group comparison. (Log2 Fold Change = Log2FC).

G1 versus G5

Entry	Entry Name UNIPROT	Protein names	Mass	Gene Names	Log2FC
C0JYZ2	C0JYZ2_HUMAN	Titin	3,713,712 Da	TTN	5,70
Q8N7U2	Q8N7U2_HUMAN	cDNA FLJ40342 fis, clone TESTI2032768	59,919 Da		5,67
V9HW34	V9HW34_HUMAN	Epididymis luminal protein 213	25,646 Da	HEL-213	5,52
P68871	HBB_HUMAN	Hemoglobin subunit beta	15,998 Da	HBB	5,39
P01037	CYTN_HUMAN	Cystatin-SN	16,388 Da	CST1	5,04
B3KW67	B3KW67_HUMAN	cDNA FLJ42347 fis, clone UTERU2003399, highly similar to Actin, gamma-enteric smooth muscle	41,817 Da		5,00
Q8WVW5	Q8WVW5_HUMAN	Actin, cytoplasmic 1	40,503 Da		5,00
Q14669	TRIPC_HUMAN	E3 ubiquitin-protein ligase TRIP12	220,434 Da	TRIP12, KIAA0045, ULF	4,81
P02647	APOA1_HUMAN	Apolipoprotein A-I	30,778 Da	APOA1	4,75
E7ERK0	E7ERK0_HUMAN	Mucin 4, cell surface associated	433,815 Da	MUC4	4,70
Q6PYX1	Q6PYX1_HUMAN	Hepatitis B virus receptor binding protein	38,162 Da		4,70
E7ETD6	E7ETD6_HUMAN	Bromodomain PHD finger transcription factor	308,073 Da	BPTF	4,70
F6KPG5	F6KPG5_HUMAN	Albumin	66,531 Da		4,64
Q8N488	RYBP_HUMAN	RING1 and YY1-binding protein	24,822 Da	RYBP, DEDAF, YEAF1	4,64
H6VRF9	H6VRF9_HUMAN	Keratin, type II cytoskeletal 1	66,069 Da	KRT1	4,39
B3KME6	B3KME6_HUMAN	cDNA FLJ10802 fis, clone NT2RP4000817, highly similar to Homo sapiens centrosomal protein 170 kDa (CEP170), transcript variant alpha, mRNA	76,231 Da		4,39
B3KXP1	B3KXP1_HUMAN	Ankyrin repeat and SAM domain-containing protein 6	72,503 Da		4,32
Q13516	OLIG2_HUMAN	Oligodendrocyte transcription factor 2	32,385 Da	OLIG2, BHLHB1, BHLHE19, PRKCBP2, RACK17	4,32

Q8NBF1	GLIS1_HUMAN	Zinc finger protein GLIS1	65,976 Da	GLIS1	4,32
Q9UKN1	MUC12_HUMAN	Mucin-12	558,164 Da	MUC12, MUC11	4,32
B4DYD1	B4DYD1_HUMAN	Sperm acrosome membrane-associated protein 3	54,767 Da		4,32
Q8TEM4	Q8TEM4_HUMAN	ADP-ribosylation factor-like protein 6-interacting protein 4	46,514 Da	FLJ00169	4,32
P00738	HPT_HUMAN	Haptoglobin	45,205 Da	HP	4,25
A4D2Q0	A4D2Q0_HUMAN	Unc-84 homolog A (C. elegans)	108,455 Da	UNC84A, tcag7.1079	4,25
H0Y6B5	H0Y6B5_HUMAN	Abl interactor 2	31,877 Da	ABI2	4,25
Q99102	MUC4_HUMAN	Mucin-4	542,307 Da	MUC4	-7,86
Q8WXI7	MUC16_HUMAN	Mucin-16	1,519,175 Da	MUC16, CA125	-6,55
Q9Y5I3	PCDA1_HUMAN	Protocadherin alpha-1	102,952 Da	PCDHA1	-5,86
A8K8M7	A8K8M7_HUMAN	cDNA FLJ76439, highly similar to Homo sapiens BTB (POZ) domain containing 10 (BTBD10), mRNA	53,765 Da		-5,32
Q6ZS56	Q6ZS56_HUMAN	cDNA FLJ45819 fis, clone NT2RP8001363, highly similar to Mus musculus signal peptide, CUB domain, EGF-like 1 (Scube1)	67,000 Da		-4,95
Q92833	JARD2_HUMAN	Protein Jumonji	138,734 Da	JARID2, JMJ	-4,91
G5E9V3	G5E9V3_HUMAN	Pleckstrin homology like domain family B member 2	64,180 Da	PHLDB2, hCG_2036872	-4,70
Q5SYB0	FRPD1_HUMAN	FERM and PDZ domain-containing protein 1	173,437 Da	FRMPD1, FRMD2, KIAA0967	-4,58
K7ER84	K7ER84_HUMAN	Podocan like 1	17,843 Da	PODNL1	-4,46
Q5VV67	PPRC1_HUMAN	Peroxisome proliferator-activated receptor gamma coactivator-related protein 1	177,544 Da	PPRC1, KIAA0595	-4,46
B7ZA42	B7ZA42_HUMAN	cDNA, FLJ79056	77,242 Da		-4,39
F4MHI3	F4MHI3_HUMAN	[histone H3]-trimethyl-L-lysine(27) demethylase	111,327 Da	UTY	-4,39
B7ZM61	B7ZM61_HUMAN	Phosphoinositide phospholipase C	257,104 Da	PLCE1	-4,39
P78333	GPC5_HUMAN	Glypican-5	63,707 Da	GPC5	-4,39

Q0KKI6	Q0KKI6_HUMAN	Immunoblobulin light chain	24,030 Da		-4,32
B1W6B2	B1W6B2_HUMAN	NADH-ubiquinone oxidoreductase chain 5	67,399 Da	NADH5, ndh5	-4,32
Q8WYL5	SSH1_HUMAN	Protein phosphatase Slingshot homolog 1	115,511 Da	SSH1, KIAA1298, SSH1L	-4,32
Q9NSI6	BRWD1_HUMAN	Bromodomain and WD repeat-containing protein 1	262,936 Da	BRWD1, C21orf107, WDR9	-4,25
Q86UP3	ZFHX4_HUMAN	Zinc finger homeobox protein 4	393,730 Da	ZFHX4	-4,25
O60307	MAST3_HUMAN	Microtubule-associated serine/threonine-protein kinase 3	143,137 Da	MAST3, KIAA0561	-4,25
Q9Y6M7	S4A7_HUMAN	Sodium bicarbonate cotransporter 3	136,044 Da	SLC4A7, BT, NBC2, NBC2B, NBC3, NBCn1, SBC2, SLC4A6	-4,25
Q7Z398	ZN550_HUMAN	Zinc finger protein 550	48,381 Da	ZNF550	-4,25
C9J444	C9J444_HUMAN	Oligodendrocyte transcription factor 2	6,147 Da	OLIG2	-4,25

G2 versus G5

Entry	Entry Name UNIPROT	Protein names	Mass	Gene Names	Log2FC
T1R7N1	T1R7N1_HUMAN	MUC5AC	342,471 Da	MUC5AC	5,93
Q9HC84	MUC5B_HUMAN	Mucin-5B	596,340 Da	MUC5B, MUC5	5,29
C9K0V9	C9K0V9_HUMAN	Ataxin 7 like 1	78,190 Da	ATXN7L1	5,13
P01037	CYTN_HUMAN	Cystatin-SN	16,388 Da	CST1	5,09
S6C4S2	S6C4S2_HUMAN	IgG L chain	22,861 Da		5,00
A8K6L7	A8K6L7_HUMAN	Rho GTPase-activating protein 7	170,581 Da		4,95
Q6PJF2	Q6PJF2_HUMAN	IGK@ protein	25,521 Da	IGK@	4,95
P41180	CASR_HUMAN	Extracellular calcium-sensing receptor	120,675 Da	CASR, GPRC2A, PCAR1	4,95
P68871	HBB_HUMAN	Hemoglobin subunit beta	15,998 Da	HBB	4,91
L8E7G9	L8E7G9_HUMAN	Alternative protein ZNF74	32,469 Da	ZNF74	4,91
Q8N2R8	FA43A_HUMAN	Protein FAM43A	45,776 Da	FAM43A, PP7298	4,86

Q70EK8	UBP53_HUMAN	Ubiquitin carboxyl-terminal hydrolase 53	120,806 Da	USP53, KIAA1350	4,81
Q2Q1W2	LIN41_HUMAN	E3 ubiquitin-protein ligase TRIM71	93,385 Da	TRIM71, LIN41	4,75
F6TH74	F6TH74_HUMAN	Phosphatase and actin regulator	18,919 Da	PHACTR2	4,70
Q96F05	CK024_HUMAN	Uncharacterized protein C11orf24	46,101 Da	C11orf24, FP2568, UNQ1872/PRO4315	4,70
Q9UKN1	MUC12_HUMAN	Mucin-12	558,164 Da	MUC12, MUC11	4,64
A4UGR9	XIRP2_HUMAN	Xin actin-binding repeat-containing protein 2	382,300 Da	XIRP2, CMYA3	4,64
X6R7H2	X6R7H2_HUMAN	Peroxisome proliferator-activated receptor gamma coactivator-related protein 1	69,098 Da	PPRC1	4,58
Q8IVL1	NAV2_HUMAN	Neuron navigator 2	268,167 Da	NAV2, HELAD1, KIAA1419, POMFIL2, RAINB1, STEERIN2	4,58
O43419	O43419_HUMAN	Intestinal mucin	60,510 Da	MUC3	4,52
Q96K68	Q96K68_HUMAN	cDNA FLJ14473 fis, clone MAMMA1001080, highly similar to Homo sapiens SNC73 protein (SNC73) mRNA	53,088 Da		4,52
Q2LD37	BLTP1_HUMAN	Bridge-like lipid transfer protein family member 1	555,482 Da	BLTP1, FSA, KIAA1109, KIAA1371, TWEEK	4,46
B7Z7H2	B7Z7H2_HUMAN	SH3 and PX domain-containing protein 2A	106,385 Da		4,46
Q5T752	LCE1D_HUMAN	Late cornified envelope protein 1D	11,230 Da	LCE1D, LEP4	4,46
Q96RG4	Q96RG4_HUMAN	Insulin receptor substrate 2	137,319 Da	IRS2	4,46
A0A087WUZ6 (deleted)	A0A087WUZ6_HUMAN	(deleted)			-6,27
Q9Y5I3	PCDA1_HUMAN	Protocadherin alpha-1	102,952 Da	PCDHA1	-5,86
P98088	MUC5A_HUMAN	Mucin-5AC	585,570 Da	MUC5AC, MUC5	-5,81
A7Y9J9	A7Y9J9_HUMAN	Mucin 5AC, oligomeric mucus/gel-forming	648,803 Da	MUC5AC	-5,75
A8K8M7	A8K8M7_HUMAN	cDNA FLJ76439, highly similar to Homo sapiens BTB (POZ) domain containing 10 (BTBD10), mRNA	53,765 Da		-5,32

Q53TD9	Q53TD9_HUMAN	Zinc finger E-box-binding homeobox 2	121,412 Da	ZFHX1B	-5,25
Q92833	JARD2_HUMAN	Protein Jumonji	138,734 Da	JARID2, JMJ	-4,91
G5E9V3	G5E9V3_HUMAN	Pleckstrin homology like domain family B member 2	64,180 Da	PHLDB2, hCG_2036872	-4,70
Q5SYB0	FRPD1_HUMAN	FERM and PDZ domain-containing protein 1	173,437 Da	FRMPD1, FRMD2, KIAA0967	-4,58
Q9UF83	YM012_HUMAN	Uncharacterized protein DKFZp434B061	59,412 Da		-4,52
K7ER84	K7ER84_HUMAN	Podocan like 1	17,843 Da	PODNL1	-4,46
Q5VV67	PPRC1_HUMAN	Peroxisome proliferator-activated receptor gamma coactivator-related protein 1	177,544 Da	PPRC1, KIAA0595	-4,46
Q02505	MUC3A_HUMAN	Mucin-3A	345,127 Da	MUC3A, MUC3	-4,39
F8W6W8	F8W6W8_HUMAN	Regulating synaptic membrane exocytosis 2	153,781 Da	RIMS2	-4,39
O15265	ATX7_HUMAN	Ataxin-7	95,451 Da	ATXN7, SCA7	-4,39
F4MHI3	F4MHI3_HUMAN	[histone H3]-trimethyl-L-lysine(27) demethylase	111,327 Da	UTY	-4,39
B7ZM61	B7ZM61_HUMAN	Phosphoinositide phospholipase C	257,104 Da	PLCE1	-4,39
P78333	GPC5_HUMAN	Glypican-5	63,707 Da	GPC5	-4,39
H0YJP0	H0YJP0_HUMAN	E3 ubiquitin-protein ligase	279,412 Da	HECTD1	-4,32
Q0KKI6	Q0KKI6_HUMAN	Immunoblobulin light chain	24,030 Da		-4,32
B1W6B2	B1W6B2_HUMAN	NADH-ubiquinone oxidoreductase chain 5	67,399 Da	NADH5, ndh5	-4,32
Q8WYL5	SSH1_HUMAN	Protein phosphatase Slingshot homolog 1	115,511 Da	SSH1, KIAA1298, SSH1L	-4,32
B2RWP5	B2RWP5_HUMAN	Histone-lysine N-methyltransferase, H3 lysine-36 specific	267,350 Da	NSD1	-4,25
O60307	MAST3_HUMAN	Microtubule-associated serine/threonine-protein kinase 3	143,137 Da	MAST3, KIAA0561	-4,25
Q9Y6M7	S4A7_HUMAN	Sodium bicarbonate cotransporter 3	136,044 Da	SLC4A7, BT, NBC2, NBC2B, NBC3, NBCn1, SBC2, SLC4A6	-4,25

G3 versus G5

Entry	Entry Name UNIPROT	Protein names	Mass	Gene Names	Log2FC
C0JYZ2	C0JYZ2_HUMAN	Titin	3,713,712 Da	TTN	5,67

Q6PJF2	Q6PJF2_HUMAN	IGK@ protein	25,521 Da	IGK@	5,32
B4DZG6	B4DZG6_HUMAN	Nucleoporin NDC1	37,928 Da		5,32
T1R7N3	T1R7N3_HUMAN	MUC5AC	413,870 Da	MUC5AC	5,25
P68871	HBB_HUMAN	Hemoglobin subunit beta	15,998 Da	HBB	5,09
P01037	CYTN_HUMAN	Cystatin-SN	16,388 Da	CST1	5,09
Q9HC84	MUC5B_HUMAN	Mucin-5B	596,340 Da	MUC5B, MUC5	5,04
F8WEP2	F8WEP2_HUMAN	ADP-ribosylation factor-like protein 6-interacting protein 4	19,132 Da	ARL6IP4	4,95
Q86UR5	RIMS1_HUMAN	Regulating synaptic membrane exocytosis protein 1	189,073 Da	RIMS1, KIAA0340, RAB3IP2, RIM1, Nbla00761	4,95
A8K964	A8K964_HUMAN	Pinin	81,542 Da		4,91
B7Z7A2	B7Z7A2_HUMAN	Phosphodiesterase	57,469 Da		4,75
X6R7H2	X6R7H2_HUMAN	Peroxisome proliferator-activated receptor gamma coactivator-related protein 1	69,098 Da	PPRC1	4,70
A4D1A8	A4D1A8_HUMAN	Similar to Piccolo protein (Aczonin)	411,161 Da	LOC392742, tcag7.1316	4,46
H7BZY0	H7BZY0_HUMAN	Tumor protein p53 binding protein 1	17,965 Da	TP53BP1	4,46
Q9Y566	SHAN1_HUMAN	SH3 and multiple ankyrin repeat domains protein 1	224,959 Da	SHANK1	4,39
F6KPG5	F6KPG5_HUMAN	Albumin	66,531 Da		4,32
P01857	IGHG1_HUMAN	Immunoglobulin heavy constant gamma 1	43,912 Da	IGHG1	4,17
O95996	APCL_HUMAN	Adenomatous polyposis coli protein 2	243,949 Da	APC2, APCL	4,09
H3BPE1	H3BPE1_HUMAN	Microtubule actin crosslinking factor 1	856,879 Da	MACF1	4,09
S6BGD6	S6BGD6_HUMAN	IgG L chain	24,824 Da		4,09
E9PDQ1	E9PDQ1_HUMAN	Alpha-S1-casein	20,821 Da	CSN1S1	4,09
J9JIF5	J9JIF5_HUMAN	CXXC-type zinc finger protein 4	36,514 Da	CXXC4	4,00
B7Z7S7	B7Z7S7_HUMAN	cDNA FLJ60964, weakly similar to Homo sapiens dentin sialophosphoprotein (DSPP), mRNA	37,696 Da		4,00

H0YF33	H0YF33_HUMAN	Nuclear autoantigenic sperm protein	31,789 Da	NASP	4,00
Q2F3K1	Q2F3K1_HUMAN	Extracellular calcium-sensing receptor	55,961 Da	CASR	4,00
Q99102	MUC4_HUMAN	Mucin-4	542,307 Da	MUC4	-7,86
Q8WXI7	MUC16_HUMAN	Mucin-16	1,519,175 Da	MUC16, CA125	-6,36
Q9Y5I3	PCDA1_HUMAN	Protocadherin alpha-1	102,952 Da	PCDHA1	-5,86
P98088	MUC5A_HUMAN	Mucin-5AC	585,570 Da	MUC5AC, MUC5	-5,81
A7Y9J9	A7Y9J9_HUMAN	Mucin 5AC, oligomeric mucus/gel-forming	648,803 Da	MUC5AC	-5,75
Q9Y6V0	PCLO_HUMAN	Protein piccolo	560,699 Da	PCLO, ACZ, KIAA0559	-5,67
Q53TD9	Q53TD9_HUMAN	Zinc finger E-box-binding homeobox 2	121,412 Da	ZFHX1B	-5,25
Q92833	JARD2_HUMAN	Protein Jumonji	138,734 Da	JARID2, JMJ	-4,91
G5E9V3	G5E9V3_HUMAN	Pleckstrin homology like domain family B member 2	64,180 Da	PHLDB2, hCG_2036872	-4,70
Q5SYB0	FRPD1_HUMAN	FERM and PDZ domain-containing protein 1	173,437 Da	FRMPD1, FRMD2, KIAA0967	-4,58
K7ER84	K7ER84_HUMAN	Podocan like 1	17,843 Da	PODNL1	-4,46
Q5VV67	PPRC1_HUMAN	Peroxisome proliferator-activated receptor gamma coactivator-related protein 1	177,544 Da	PPRC1, KIAA0595	-4,46
B7ZA42	B7ZA42_HUMAN	cDNA, FLJ79056	77,242 Da		-4,39
Q02505	MUC3A_HUMAN	Mucin-3A	345,127 Da	MUC3A, MUC3	-4,39
F4MHI3	F4MHI3_HUMAN	[histone H3]-trimethyl-L-lysine(27) demethylase	111,327 Da	UTY	-4,39
B7ZM61	B7ZM61_HUMAN	Phosphoinositide phospholipase C	257,104 Da	PLCE1	-4,39
P78333	GPC5_HUMAN	Glypican-5	63,707 Da	GPC5	-4,39
H0YJP0	H0YJP0_HUMAN	E3 ubiquitin-protein ligase	279,412 Da	HECTD1	-4,32
Q0KKI6	Q0KKI6_HUMAN	Immunoblobulin light chain	24,030 Da		-4,32
B1W6B2	B1W6B2_HUMAN	NADH-ubiquinone oxidoreductase chain 5	67,399 Da	NADH5, ndh5	-4,32
Q8WYL5	SSH1_HUMAN	Protein phosphatase Slingshot homolog 1	115,511 Da	SSH1, KIAA1298, SSH1L	-4,32
Q9NSI6	BRWD1_HUMAN	Bromodomain and WD repeat-containing protein 1	262,936 Da	BRWD1, C21orf107, WDR9	-4,25

B2RWP5	B2RWP5_HUMAN	Histone-lysine N-methyltransferase, H3 lysine-36 specific	267,350 Da	NSD1	-4,25
O60307	MAST3_HUMAN	Microtubule-associated serine/threonine-protein kinase 3	143,137 Da	MAST3, KIAA0561	-4,25
Q9Y6M7	S4A7_HUMAN	Sodium bicarbonate cotransporter 3	136,044 Da	SLC4A7, BT, NBC2, NBC2B, NBC3, NBCn1, SBC2, SLC4A6	-4,25

G4 versus G5

Entry	Entry Name UNIPROT	Protein names	Mass	Gene Names	Log2FC
Q86UR5	RIMS1_HUMAN	Regulating synaptic membrane exocytosis protein 1	189,073 Da	RIMS1, KIAA0340, RAB3IP2, RIM1, Nbla00761	5,83
B4DZG6	B4DZG6_HUMAN	Nucleoporin NDC1	37,928 Da		5,67
Q9HC84	MUC5B_HUMAN	Mucin-5B	596,340 Da	MUC5B, MUC5	5,64
C8C504	C8C504_HUMAN	Beta-globin	15,997 Da	HBB	5,52
T1S9D5	T1S9D5_HUMAN	MUC5AC	521,632 Da	MUC5AC	5,49
Q6PJF2	Q6PJF2_HUMAN	IGK@ protein	25,521 Da	IGK@	5,39
T1R7N3	T1R7N3_HUMAN	MUC5AC	413,870 Da	MUC5AC	5,32
E7EQG8	E7EQG8_HUMAN	Mucin 4, cell surface associated	432,659 Da	MUC4	5,13
Q96HP0	DOCK6_HUMAN	Dedicator of cytokinesis protein 6	229,558 Da	DOCK6, KIAA1395	5,04
B3KW67	B3KW67_HUMAN	cDNA FLJ42347 fis, clone UTERU2003399, highly similar to Actin, gamma-enteric smooth muscle	41,817 Da		4,75
Q6PRX2	Q6PRX2_HUMAN	Transducin-like enhancer of split 3 splice variant 2	82,883 Da	TLE3	4,64
H3BUG0	H3BUG0_HUMAN	RNA binding protein with serine rich domain 1	14,634 Da	RNPS1	4,52
B3KNX8	B3KNX8_HUMAN	cDNA FLJ30689 fis, clone FCBBF2000566, highly similar to B-cell lymphoma/leukemia 11A	84,504 Da		4,52
B7ZKN7	B7ZKN7_HUMAN	RecQ-like DNA helicase BLM	117,063 Da	BLM	4,46
H0YKT6	H0YKT6_HUMAN	Aminopeptidase N	11,099 Da	ANPEP	4,46
F6KPG5	F6KPG5_HUMAN	Albumin	66,531 Da		4,39
Q92766	RREB1_HUMAN	Ras-responsive element-binding protein 1	181,420 Da	RREB1, FINB	4,39

A4D1R9	A4D1R9_HUMAN	Homeodomain interacting protein kinase 2	88,647 Da	HIPK2, tcag7.426	4,32
A0A0C4DG40	A0A0C4DG40_HUMAN	Nesprin-1	1,005,181 Da	SYNE1	4,32
P48169	GBRA4_HUMAN	Gamma-aminobutyric acid receptor subunit alpha-4	61,623 Da	GABRA4	4,32
H0Y9R9	H0Y9R9_HUMAN	Alpha-L-iduronidase	21,924 Da	IDUA	4,32
Q15413	RYR3_HUMAN	Ryanodine receptor 3	552,042 Da	RYR3, HBRR	4,25
B4DFU0	B4DFU0_HUMAN	Protein lin-54 homolog	75,247 Da		4,25
A0A0A0MR84	A0A0A0MR84_HUMAN	Zinc finger protein 446	36,309 Da	ZNF446	4,25
L8ECC4	L8ECC4_HUMAN	Alternative protein MAFB	17,635 Da	MAFB	4,25
Q99102	MUC4_HUMAN	Mucin-4	542,307 Da	MUC4	-7,86
Q8WXI7	MUC16_HUMAN	Mucin-16	1,519,175 Da	MUC16, CA125	-6,36
Q9Y5I3	PCDA1_HUMAN	Protocadherin alpha-1	102,952 Da	PCDHA1	-5,86
P98088	MUC5A_HUMAN	Mucin-5AC	585,570 Da	MUC5AC, MUC5	-5,81
A7Y9J9	A7Y9J9_HUMAN	Mucin 5AC, oligomeric mucus/gel-forming	648,803 Da	MUC5AC	-5,75
Q6ZS56	Q6ZS56_HUMAN	cDNA FLJ45819 fis, clone NT2RP8001363, highly similar to Mus musculus signal peptide, CUB domain, EGF-like 1 (Scube1)	67,000 Da		-4,95
Q92833	JARD2_HUMAN	Protein Jumonji	138,734 Da	JARID2, JMJ	-4,91
G5E9V3	G5E9V3_HUMAN	Pleckstrin homology like domain family B member 2	64,180 Da	PHLDB2, hCG_2036872	-4,70
Q5SYB0	FRPD1_HUMAN	FERM and PDZ domain-containing protein 1	173,437 Da	FRMPD1, FRMD2, KIAA0967	-4,58
K7ER84	K7ER84_HUMAN	Podocan like 1	17,843 Da	PODNL1	-4,46
Q5VV67	PPRC1_HUMAN	Peroxisome proliferator-activated receptor gamma coactivator-related protein 1	177,544 Da	PPRC1, KIAA0595	-4,46
F8W6W8	F8W6W8_HUMAN	Regulating synaptic membrane exocytosis 2	153,781 Da	RIMS2	-4,39
O15265	ATX7_HUMAN	Ataxin-7	95,451 Da	ATXN7, SCA7	-4,39
F4MHI3	F4MHI3_HUMAN	[histone H3]-trimethyl-L-lysine(27) demethylase	111,327 Da	UTY	-4,39

B7ZM61	B7ZM61_HUMAN	Phosphoinositide phospholipase C	257,104 Da	PLCE1	-4,39
P78333	GPC5_HUMAN	Glypican-5	63,707 Da	GPC5	-4,39
H0YJP0	H0YJP0_HUMAN	E3 ubiquitin-protein ligase	279,412 Da	HECTD1	-4,32
Q0KKI6	Q0KKI6_HUMAN	Immunoblobulin light chain	24,030 Da		-4,32
B1W6B2	B1W6B2_HUMAN	NADH-ubiquinone oxidoreductase chain 5	67,399 Da	NADH5, ndh5	-4,32
Q8WYL5	SSH1_HUMAN	Protein phosphatase Slingshot homolog 1	115,511 Da	SSH1, KIAA1298, SSH1L	-4,32
Q86UP3	ZFHX4_HUMAN	Zinc finger homeobox protein 4	393,730 Da	ZFHX4	-4,25
B2RWP5	B2RWP5_HUMAN	Histone-lysine N-methyltransferase, H3 lysine-36 specific	267,350 Da	NSD1	-4,25
Q9Y6M7	S4A7_HUMAN	Sodium bicarbonate cotransporter 3	136,044 Da	SLC4A7, BT, NBC2, NBC2B, NBC3, NBCn1, SBC2, SLC4A6	-4,25
Q7Z398	ZN550_HUMAN	Zinc finger protein 550	48,381 Da	ZNF550	-4,25
C9J444	C9J444_HUMAN	Oligodendrocyte transcription factor 2	6,147 Da	OLIG2	-4,25

Supplementary Figure S2. Pathway and process enrichment analyses of up-regulated differentially abundant proteins of diseased group compared with control group (Metascape.org platform).

T2DMpoorly-DL-P versus Control								T2DMwell-DL-P versus Control						
**GO**	**Category**	**Description**	**Count**	**%**	**Log10 (P)**	**Log10 (q)**		**GO**	**Category**	**Description**	**Count**	**%**	**Log10 (P)**	**Log10 (q)**
WP4629	WikiPathways	Aerobic glycolysis	5	3.25	-8.61	-4.36		GO:0034330	GO Biological Processes	cell junction organization	18	7.38	-6.03	-1.75
R-HSA-2168880	Reactome Gene Sets	Scavenging of heme from plasma	4	2.6	-6.69	-3.09		GO:0030036	GO Biological Processes	actin cytoskeleton organization	17	6.97	-5.6	-1.71
R-HSA-114608	Reactome Gene Sets	Platelet degranulation	7	4.55	-5.94	-2.71		GO:0050905	GO Biological Processes	neuromuscular process	9	3.69	-5.21	-1.53
GO:0051651	GO Biological Processes	maintenance of location in cell	6	3.9	-5.03	-2.19		GO:0006259	GO Biological Processes	DNA metabolic process	19	7.79	-4.93	-1.35
WP4656	WikiPathways	Joubert syndrome	5	3.25	-4.36	-1.66		GO:0007610	GO Biological Processes	behavior	17	6.97	-4.76	-1.27
GO:0009792	GO Biological Processes	embryo development ending in birth or egg hatching	13	8.44	-4.32	-1.65		hsa04934	KEGG Pathway	Cushing syndrome	8	3.28	-4.4	-1.03
GO:0097435	GO Biological Processes	supramolecular fiber organization	12	7.79	-4.16	-1.52		GO:0048729	GO Biological Processes	tissue morphogenesis	15	6.15	-4.09	-0.77
GO:0120031	GO Biological Processes	plasma membrane bounded cell projection assembly	10	6.49	-4.05	-1.45		GO:0060134	GO Biological Processes	prepulse inhibition	3	1.23	-3.86	-0.72
R-HSA-9619665	Reactome Gene Sets	EGR2 and SOX10-mediated initiation of Schwann cell myelination	3	1.95	-3.67	-1.16		GO:0022604	GO Biological Processes	regulation of cell morphogenesis	9	3.69	-3.72	-0.64
GO:0010954	GO Biological Processes	positive regulation of protein processing	3	1.95	-3.56	-1.07		GO:0032102	GO Biological Processes	negative regulation of response to external stimulus	12	4.92	-3.54	-0.54
GO:0035994	GO Biological Processes	response to muscle stretch	3	1.95	-3.56	-1.07		WP4148	WikiPathways	Splicing factor NOVA regulated synaptic proteins	4	1.64	-3.44	-0.52
GO:1900024	GO Biological Processes	regulation of substrate adhesion-dependent cell spreading	4	2.6	-3.54	-1.06		GO:1903729	GO Biological Processes	regulation of plasma membrane organization	3	1.23	-3.41	-0.52
CORUM:5386	CORUM	MLL1-WDR5 complex	3	1.95	-3.46	-1.01		WP5360	WikiPathways	KCNQ2 related epilepsies	3	1.23	-3.34	-0.49
M3008	Canonical Pathways	NABA ECM GLYCOPROTEINS	6	3.9	-3.29	-0.87		GO:1903828	GO Biological Processes	negative regulation of protein localization	8	3.28	-3.28	-0.46
GO:1905475	GO Biological Processes	regulation of protein localization to membrane	6	3.9	-3.27	-0.87		GO:0006351	GO Biological Processes	DNA-templated transcription	13	5.33	-3.24	-0.45
GO:2000649	GO Biological Processes	regulation of sodium ion transmembrane transporter activity	3	1.95	-3.24	-0.85		GO:0140694	GO Biological Processes	membraneless organelle assembly	10	4.1	-3.21	-0.45
GO:0007389	GO Biological Processes	pattern specification process	9	5.84	-3.21	-0.83		GO:0031344	GO Biological Processes	regulation of cell projection organization	14	5.74	-2.95	-0.33
GO:0120032	GO Biological Processes	regulation of plasma membrane bounded cell projection assembly	6	3.9	-3.17	-0.8		WP2853	WikiPathways	Endoderm differentiation	6	2.46	-2.94	-0.33
GO:0010804	GO Biological Processes	negative regulation of tumor necrosis factor-mediated signaling pathway	3	1.95	-3.16	-0.79		GO:1903047	GO Biological Processes	mitotic cell cycle process	12	4.92	-2.93	-0.32
M223	Canonical Pathways	PID BETA CATENIN NUC PATHWAY	4	2.6	-3.12	-0.77		GO:0051057	GO Biological Processes	positive regulation of small GTPase mediated signal transduction	4	1.64	-2.65	-0.22

continues

continuation of Figure S2. Pathway and enrichment analyses of up-regulated differentially abundant proteins of a diseased group compared with the control group (Metascape.org platform).

DL-P versus Control								P versus Control						
GO	Category	Description	Count	%	Log10 (P)	Log10 (q)		GO	Category	Description	Count	%	Log10 (P)	Log10 (q)
GO:0010639	GO Biological Processes	negative regulation of organelle organization	10	8.62	-6.01	-1.7		GO:0034330	GO Biological Processes	cell junction organization	17	10.9	-8.19	-4.05
GO:1902115	GO Biological Processes	regulation of organelle assembly	8	6.9	-5.43	-1.7		GO:0051493	GO Biological Processes	regulation of cytoskeleton organization	14	8.97	-6.07	-2.2
R-HSA-5083625	Reactome Gene Sets	Defective GALNT3 causes HFTC	3	2.59	-4.45	-1.09		GO:1902903	GO Biological Processes	regulation of supramolecular fiber organization	12	7.69	-5.89	-2.15
GO:0009166	GO Biological Processes	nucleotide catabolic process	5	4.31	-4.12	-0.93		R-HSA-3906995	Reactome Gene Sets	Diseases associated with O-glycosylation of proteins	5	3.21	-4.69	-1.3
GO:0036092	GO Biological Processes	phosphatidylinositol-3-phosphate biosynthetic process	3	2.59	-4.04	-0.92		GO:1901990	GO Biological Processes	regulation of mitotic cell cycle phase transition	10	6.41	-4.61	-1.27
hsa05022	KEGG Pathway	Pathways of neurodegeneration - multiple diseases	9	7.76	-3.98	-0.92		GO:2000651	GO Biological Processes	positive regulation of sodium ion transmembrane transporter activity	3	1.92	-4.55	-1.26
GO:0098754	GO Biological Processes	detoxification	5	4.31	-3.55	-0.68		GO:0048738	GO Biological Processes	cardiac muscle tissue development	7	4.49	-4.12	-1
R-HSA-2173782	Reactome Gene Sets	Binding and Uptake of Ligands by Scavenger Receptors	3	2.59	-3.48	-0.65		GO:1901976	GO Biological Processes	regulation of cell cycle checkpoint	4	2.56	-3.92	-0.94
M68	Canonical Pathways	PID RHOA REG PATHWAY	3	2.59	-3.13	-0.49		GO:0032922	GO Biological Processes	circadian regulation of gene expression	4	2.56	-3.39	-0.57
GO:0001824	GO Biological Processes	blastocyst development	4	3.45	-3.02	-0.43		WP2361	WikiPathways	Gastric cancer network 1	3	1.92	-3.35	-0.56
GO:0031644	GO Biological Processes	regulation of nervous system process	4	3.45	-2.87	-0.31		GO:0071479	GO Biological Processes	cellular response to ionizing radiation	4	2.56	-3.23	-0.51
GO:0140029	GO Biological Processes	exocytic process	3	2.59	-2.76	-0.22		R-HSA-1474244	Reactome Gene Sets	Extracellular matrix organization	7	4.49	-3.18	-0.48
WP3680	WikiPathways	Physico chemical features and toxicity associated pathways	3	2.59	-2.68	-0.18		GO:0042325	GO Biological Processes	regulation of phosphorylation	11	7.05	-3.01	-0.4
WP3584	WikiPathways	MECP2 and associated Rett syndrome	3	2.59	-2.54	-0.08		WP4829	WikiPathways	mBDNF and proBDNF regulation of GABA neurotransmission	3	1.92	-3	-0.4
GO:2000736	GO Biological Processes	regulation of stem cell differentiation	3	2.59	-2.38	0		GO:0002285	GO Biological Processes	lymphocyte activation involved in immune response	5	3.21	-3	-0.4
R-HSA-109582	Reactome Gene Sets	Hemostasis	7	6.03	-2.34	0		R-HSA-983231	Reactome Gene Sets	Factors involved in megakaryocyte development and platelet production	5	3.21	-2.96	-0.38
GO:0007611	GO Biological Processes	learning or memory	5	4.31	-2.33	0		hsa04610	KEGG Pathway	Complement and coagulation cascades	4	2.56	-2.95	-0.37
GO:0016055	GO Biological Processes	Wnt signaling pathway	5	4.31	-2.32	0		GO:0001708	GO Biological Processes	cell fate specification	4	2.56	-2.91	-0.35
WP5475	WikiPathways	Hallmark of cancer sustaining proliferative signaling	3	2.59	-2.19	0		GO:0001558	GO Biological Processes	regulation of cell growth	8	5.13	-2.87	-0.33
GO:0035264	GO Biological Processes	multicellular organism growth	3	2.59	-2.18	0		GO:0007528	GO Biological Processes	neuromuscular junction development	3	1.92	-2.85	-0.32

Enrichment analyses carried out with GO Biological Processes, PANTHER Pathway, KEGG Pathway, Reactome Gene Sets, WikiPathways, Canonical Pathways, and CORUM.
